# Nutritional strategies in supporting immune checkpoint inhibitor, PI3K inhibitor, and tyrosine kinase inhibitor cancer therapies

**DOI:** 10.3389/fnut.2025.1670598

**Published:** 2025-12-03

**Authors:** Nina Fuller-Shavel, Emma Jane Davies, Shira Peleg Hasson

**Affiliations:** 1 Department of Integrative Cancer Care, Synthesis Clinic, Reading, Berkshire, United Kingdom; 2 National Centre for Integrative Oncology, Reading, Berkshire, United Kingdom

**Keywords:** immune checkpoint, cancer immunotherapy, cancer nutrition, gut microbiome, PI3K inhibitor, targeted cancer therapy, precision nutrition, integrative oncology

## Abstract

Nutritional status of patients undergoing cancer treatment has been associated with cancer therapy and survival outcomes across multiple therapy types. Targeted therapies, including immune checkpoint inhibitors (ICIs), phosphatidylinositol 3-kinase (PI3K) inhibitors and EGFR-tyrosine kinase inhibitors (TKIs), are both influenced by and themselves influence the patients’ nutritional and metabolic status. Precision nutrition approaches that address specific aspects of targeted therapies, from minimizing toxicities and treatment resistance to potential therapeutic synergies, offer an important avenue to optimize clinical outcomes for patients receiving targeted oncological treatments as a part of an overall precision integrative oncology approach. Optimizing ICI treatment may necessitate gastrointestinal microbiome modulation and managing systemic inflammation with a variety of dietary approaches under study, including the Mediterranean diet, increasing fiber and fermented food intake, fasting and fasting mimicking diet and the ketogenic diet. Supplementation approaches using live biotherapeutics alongside ICIs predominate over prebiotic, postbiotic and synbiotic studies, which require further attention and investment, alongside human research on mycotherapy and fucoidan-based combinations. Optimizing PI3K treatment tolerance requires close attention to monitoring and managing glycemic control through nutrition, lifestyle and pharmacological intervention as necessary, and in supporting patients with EGFR-TKIs both nutritional prehabilitation and close attention to managing gastrointestinal toxicities is paramount. Rational individualized approaches based on detailed and dynamic clinical assessment of patient-, cancer- and treatment-related factors, using validated prognostic scores and biomarkers, are needed to maximize the potential of precision nutrition now and in future trials in this arena.

## 1 Introduction

The expansion of access to next-generation sequencing (NGS) for detailed tumor profiling alongside targeted oncological therapies and immunotherapy ushered in the era of precision oncology. Precision oncology aims to provide the most clinically effective, personalized and dynamically adjusted treatment strategy based on the profile of the patient’s cancer, including its spatial and temporal heterogeneity, and patient characteristics (1). The application of precision oncology is often seen to be specifically centered around tumor profiling to support early diagnosis, treatment selection, assessment of response and treatment resistance. However, it is becoming increasingly important to view tumor-derived data within a broader systems and patient context, such as considering the influence of the gastrointestinal (GI) microbiome and systemic inflammation on the effectiveness of immune checkpoint inhibitor (ICI) therapies (2–4).

This narrative review aims to review key nutritional strategies in supporting targeted cancer therapies with a sequential focus on immune checkpoint inhibitor, PI3K inhibitor and EGFR-TKI treatments. These targeted therapies are both influenced by and themselves influence the patients’ nutritional and metabolic status. Precision nutrition approaches aimed at minimizing toxicities and treatment resistance and encouraging potential therapeutic synergies offer an important avenue to optimize clinical outcomes for patients receiving these targeted therapies as a part of the overall precision integrative oncology care plan.

### 1.1 Introduction to immune checkpoint inhibitors

Immune checkpoints are negative regulators of the immune response that aim to promote self-tolerance and appropriately modulate normal physiological mobilization to limit tissue damage. Tumors can exploit these checkpoints as one of the ways to evade effector T cell-mediated killing. For example, CTLA-4 (cytotoxic T-lymphocyte associated protein 4) on antigen-presenting cells (APCs) binds to T cells with a higher affinity than the co-stimulatory CD28 signal, reducing T cell activation and weakening the anti-neoplastic immune response (5).

Immune checkpoint inhibition/blockade of the relevant receptor-ligand interactions using monoclonal antibodies has been explored in the treatment of a wide range of tumors with varying degrees of success from impressive results with metastatic melanoma to little net benefit in high grade serous ovarian carcinoma (HGSOC) when used as a monotherapy (6). The blocking action of immune checkpoint inhibitors takes advantage of specific ligand-receptor interaction, such as PD-L1/PD-1 (programed death-ligand 1/programed cell death protein 1), relieving inhibition of T-cell proliferation, cytokine production and cytotoxic function (5).

ICIs commonly used in solid tumor treatment include: *PD-1 inhibitors*, such as pembrolizumab and nivolumab; *PD-L1 inhibitors*, such as atezolizumab, avelumab and durvalumab; *CTLA-4 inhibitors*, such as tremelimumab and ipilimumab; and *LAG-3 (lymphocyte-activation gene 3) inhibitors*, such as relatlimab. Other immune checkpoint inhibitor therapies are in active exploration, including ICIs targeting T cell immunoglobulin and mucin-domain containing 3 (TIM3), T cell immunoglobulin and ITIM domain (TIGIT) and further anti-LAG3 therapies (7).

### 1.2 Introduction to PI3K inhibitors and EGFR-TKIs—mechanisms and applications

Elevated PI3K signaling is recognized as a hallmark of cancer (8). Activating mutations in the PIK3CA gene are frequently found across various malignancies, including breast, endometrial, cervical, colorectal, non-small cell lung, ovarian and gastric cancers (9). These mutations lead to constitutive activation of the PI3K/AKT/mTOR signaling pathway, driving oncogenesis alongside modulating cellular metabolism and immune system function. Several therapeutic strategies are available on the market to target this signaling pathway, including PI3K inhibitors, AKT inhibitors and mTOR inhibitors. All three classes of drugs are currently in clinical use in ER + (estrogen receptor positive)/HR + (hormone receptor positive) breast cancer alongside endocrine therapy (10). Clinical use of targeted PI3K and AKT inhibitors relies on a relevant documented driver mutation found on tumor profiling (10, 11).

PI3K inhibitors are associated with a distinctive spectrum of toxicities, many of which reflect immune-mediated mechanisms. The selective agents (idelalisib, duvelisib) are particularly prone to autoimmune manifestations including hepatitis, colitis, and pneumonitis. Hepatotoxicity typically presents as early transaminase elevations (often within 2–12 weeks), is frequently grade ≥ 3, and responds to corticosteroids, supporting an immune basis (12). Gastrointestinal toxicity is characterized by 2 patterns: an early, milder diarrhea and a later-onset, severe colitis that is steroid-responsive and associated with cytomegalovirus reactivation (13). Non-infectious pneumonitis, although less common, can be life-threatening. In contrast, the PI3Kα-predominant inhibitor copanlisib is primarily associated with transient metabolic adverse effects, notably infusion-related hyperglycemia and hypertension, rather than overt autoimmune complications (14).

Inhibitors of PI3Kα, most notably alpelisib, have transformed the management of hormone receptor–positive, HER2-negative, PIK3CA-mutated advanced breast cancer, but their use is frequently limited by treatment-emergent hyperglycemia. In the pivotal SOLAR-1 trial, hyperglycemia was reported in over 60% of patients and was grade 3–4 in more than one-third, making it the most common adverse event (15). This toxicity is mechanistically linked to blockade of insulin-mediated PI3K signaling, which impairs glucose uptake in skeletal muscle and adipose tissue and enhances hepatic gluconeogenesis, leading to acute insulin resistance and compensatory hyperinsulinemia (16).

Moving on to another class of targeted therapies, EGFR-tyrosine kinase inhibitors (TKIs) are small-molecule agents that bind to the intracellular catalytic domain of the epidermal growth factor receptor (EGFR), preventing it from phosphorylating and activating downstream signaling cascades involved in cancer cell proliferation and survival (17). EGFR-TKIs have opened a crucial therapeutic avenue for EGFR-sensitive mutated non-small cell lung cancer (NSCLC) but evolution of resistance limits longer term effectiveness of these treatments, with multiple combination treatments and follow-on therapy options being explored (18).

## 2 Nutrition and natural products in supporting immune checkpoint inhibitor treatment

With the rise of ICI-based regimes across multiple solid tumor indications, there has been continued increased interest in maximizing their therapeutic impact, addressing resistance to treatment and managing toxicities, such as immune-related adverse events (irAEs), which can be lifelong. A broad overview of multimodal approaches in supporting ICI treatment of solid tumors has been published previously (2), providing a foundation for a synergistic IO^2^ approach [Immuno-Oncology meets Integrative Oncology (19)]. Integrative Oncology as previously defined is “a patient-centered, evidence-informed field of cancer care that utilizes mind and body practices, natural products, and/or lifestyle modifications from different traditions alongside conventional cancer treatments. Integrative oncology aims to optimize health, quality of life, and clinical outcomes across the cancer care continuum and to empower people to prevent cancer and become active participants before, during, and beyond cancer treatment” (19).

The IO^2^ model is constantly evolving as evidence emerges and seeks to support better immunotherapy outcomes through utilizing appropriate evidence-informed integrative oncology interventions alongside standard care, including but not limited to nutrition and lifestyle modification and natural products relevant to this publication. This narrative review aims to specifically update and expand on the nutritional strategies and natural product research in ICI-based solid tumor immunotherapy.

### 2.1 Dietary approaches alongside immunotherapy with immune checkpoint inhibitors

Based on previous research, the baseline of dietary interventions in ICI support has so far been focused on a Mediterranean-style diet with at least 20 g of fiber daily, with both of these approaches being associated with improved progression-free survival (PFS) in patients with advanced melanoma receiving ICI-based treatment (4, 20). Pragmatically most clinicians choose to see 20 g as a minimum threshold and tend to follow government guidelines for fiber intake, which in the UK amounts to 30 g of fiber daily for adults. An increased daily fiber target of 30–50 g (as tolerated) may be appropriate based on the recent ASCO 2025 report of the phase 2 DIET study showing preliminary positive impact of a high fiber diet vs. 20 g fiber on survival outcomes and irAE rates with full publication awaited for further evaluation (21). A recent systematic review has highlighted the consistent association between high vs. low fiber intake and improved ICI response with a pooled OR of 5.79 demonstrated in prospective cohort studies (22). There is emerging retrospective data on fiber intake being negatively associated with ICI-induced colitis that requires prospective validation (23), alongside the new data from the DIET trial emerging above (21).

Available evidence also suggests minimization of high saturated fat and ultra-processed foods (UPFs; variably defined globally, including NOVA group 4) within the diet (24), with a recent analysis in NSCLC showing association of excessive consumption of cholesterol, sodium and saturated fat with hyperprogressive disease on ICI therapy (25). Following a wholefood-based polyphenol-rich dietary pattern, such as the Mediterranean diet, with high fiber intake and reduction in UPF and saturated fat consumption likely mediates ICI benefits through engendering a favorable GI microbiota pattern, characterized by healthy levels of Ruminococcaceae and *Bifidobacteria* spp., certain *Lachnospiraceae* spp., *Faecalibacterium prausnitzii*, and *Akkermansia muciniphila* as some examples (22, 26, 27). Additionally, this dietary pattern is known to impact short chain fatty acid (SCFA) metabolism, modulate gastrointestinal barrier integrity and function and lead to immunomodulatory effects that reduce systemic inflammation, as well as enhancing effector T cell and NK cell activity (28–33). Extensive details of the mechanisms of how gastrointestinal microbiota and the gut-immune axis influence ICI therapy have been described elsewhere (27, 34–38). As one example, high fiber wholefood-based diet enhancement of butyrate production by the gastrointestinal microbiota may lead to enhanced CD8 + effector T cell anti-tumor responses and potential synergy with ICIs through a diversity of mechanisms, including butyrate induction of the IL-12 signaling pathway, increased CD8 T cell NFkB signaling through butyrate binding to the toll-like receptor 5 (TLR5) receptor, and upregulation of PD-1 and PD-L1 expression for ICI targeting through butyrate’s action as an HDAC (histone deacetylase) inhibitor and PI3K/AKT pathway modulation (31, 39–42).

Differential microbiome patterns have been associated with response to anti-PD-1/PD-L1 ICIs compared to anti-CTLA4 therapies (43), offering a potential route to personalization that requires further study. Understanding key patterns relevant to specific ICI regimes and associated dietary or supplemental modulation strategies opens a route for intervention. Future clinical practice may aim to assess and address clinically significant patterns of baseline dysbiosis in individuals, modulating the ecosystem to achieve eubiosis associated with more favorable prognosis, ideally starting in the prehabilitation setting (44). Here TOPOSCORE, either as the original shotgun metagenomic sequencing microbiota signature-based dysbiosis scoring model or as a qPCR version more suitable for clinical practice (45), may play a role as a potential tool in both investigational and clinical settings. TOPOSCORE was derived from the combination of the ratio of SIG1 (37 bacteria associated with poor responses to PD-1 blockade in advanced NSCLC) to SIG2 (45 bacteria associated with good response), alongside *Akkermansia muciniphila* (*Akk*) abundance, with the aim of estimating the likelihood of an individual to respond to PD1/PD-L1 blockade based on the presence and degree of gastrointestinal dysbiosis (45). While TOPOSCORE needs to be studied in prospective trials, preliminary data from the algorithm incorporating these species-interacting groups (SIGs) and tripartite quantification of *Akkermansia* species provided robust predictions for overall survival in patients with renal and lung cancer SIG1, although less so for patients with melanoma (45). Interestingly, broader considerations around intratumoral microbiome and site-specific microbiome patterns, such as skin for melanoma and lung for NSCLC, and their respective interactions with the GI microbiome may also play a role in ICI therapy in the future (46–50). Looking beyond bacteria, the GI and tumor-specific mycobiome and virome also require further research and assessment for clinical contribution to neoplasia and response to treatment, including potentially ICI therapy (51, 52). With the aim of modifying the wider microbiome picture, there is a wide variety of FMT (fecal microbiome transplant) trials ongoing worldwide, such as NCT04951583, NCT04758507, NCT04729322, NCT05286294, and others summarized elsewhere (44), while some of the nutritional and supplementation avenues below would benefit from more focused attention and funding to create a diverse therapeutic toolkit applicable to a wide variety of clinical settings.

#### 2.1.1 Current interventional trials of nutritional approaches for patients on ICI immunotherapy

Moving on from retrospective nutrition studies described above, the focus for nutritional interventions in ICI support now appropriately lies in prospective studies, with some key directions and selected trials highlighted below and summarized further in Table 1:

**TABLE 1:** Overview of available and emerging clinical evidence on common nutritional and supplemental interventions targeted at microbiome modulation in supporting ICI (immune checkpoint inhibitor) treatment.

Intervention	Observational evidence and retrospective analyses	Case-based or small pilot interventional data	Preliminary interventional trial reports or phase I data	Completed and reported interventional trials (phase II-III)	Summary
**Nutritional interventions**					
Mediterranean diet	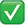	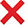	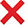	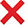	Positive linear associations between a Mediterranean dietary pattern and the probability of ORR and PFS-12 in patients receiving ICIs for advanced melanoma have been demonstrated (20) and awaits confirmation in interventional trials, such as the MINI-MD trial (NCT06236360).
Increased fiber intake and/or fiber supplementation	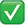	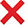	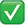	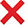	Observational evidence of an association between better PFS in patients receiving ICIs for advanced melanoma who consume at least 20 g fiber (4) has been recently followed up by an ASCO 2025 report of the phase 2 DIET study showing a preliminary positive impact trend for EFS, ORR and irAE rates with a high fiber diet (30 g escalated to 50 g daily) vs. 20 g fiber intake (21), although the data is immature and full publication is awaited for further evaluation. Several trials are ongoing, such as NCT05805319, NCT04645680, and NCT05832606.
Dietary enrichment with prebiotic-containing foods	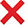	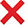	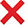	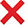	A trial of prebiotic food-enriched diet (PreFED) in ICI-refractory melanoma patients on ipilimumab and nivolumab is ongoing (NCT06250335).
Dietary enrichment with fermented foods	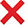	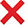	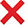	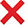	A trial of 3–6 servings of fermented foods per day in patients with stage II-IV NSCLC and locally advanced rectal cancer (NCT06337552) is ongoing, alongside a cross-over trial of fermented foods and fiber supplementation in patients with melanoma and NSCLC (NCT06475807).
Fasting regimes, including intermittent fasting (IF) and time-restricted eating (TRE)	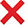	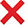	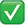	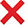	A preliminary trial report from NCT05083416 studying prolonged nightly fasting (PNF) of 14 h as a type of TRE showed that this intervention was associated with significant improvement in DCR at 3 and 6 months in treatment naïve metastatic HNSCC patients treated with single agent pembrolizumab (59), although full publication at data maturity is awaited. Several other fasting studies are ongoing, such NCT04387084 and NCT06603155.
Fasting mimicking diet (FMD)	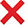	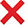	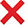	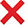	Several FMD trials have been initiated, including an FMD-ICI trial examining a 4-day FMD in advanced cancer patients on anti-PD-1/PD-L1 or anti-CTLA4 ICIs, alone or in combination (NCT06438588), and NCT06671613 for plant-based FMD in patients with metastatic NSCLC undergoing pembrolizumab treatment.
Ketogenic diet (KD)	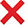	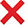	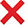	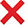	A trial of a ketogenic diet in patients with metastatic melanoma and renal cancer is ongoing (NCT06391099). One trial has been recently terminated (NCT05119010) due to multiple factors.
**Prebiotics and live biotherapeutic products**					
**Prebiotics and foods containing substances with defined or potential prebiotic effects**					
Inulin	N/A	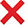	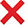	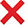	The PRINCESS study of inulin in metastatic HNSCC is ongoing (NCT05821751).
Resistant starch	N/A	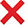	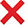	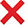	A trial of resistant potato starch supplementation in patients with solid tumors on dual ICIs is ongoing (NCT04552418).
Camu camu	N/A	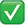	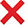	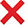	Two camu camu trials as a polyphenol-rich supplement in patients with NSCLC, melanoma and mRCC are ongoing (NCT05303493, NCT06049576).
Common single strain live biotherapeutic products (LBPs)*					
*Clostridium butyricum* CBM 588	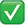	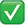	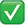	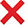	Retrospective analyses show OS benefit in NSCLC patients receiving chemoimmunotherapy, as well as improved OS for those who have received PPIs or antibiotics (67, 68). Combination data in metastatic RCC (mRCC) patients treated with cabozantinib and nivolumab showed improved PFS and ORR as a preliminary signal (69), and long-term follow-up presentation at ASCO 2025 for CBM588 with nivolumab/ipilimumab in mRCC showed improved ORR, PFS and OS (70), although full publication is awaited. Product availability may limit clinical use. Ongoing research includes CBM588 in mRCC in combination with nivolumab and cabozantinib (NCT05122546), phase I dose escalation trial of CBM588 in combination with nivolumab/ipilimumab in mRCC patients (NCT06399419) and assessment of recurrence after surgery for RCC patients with CBM588 with pembrolizumab (NCT07037004).
*Akkermansia muciniphila*, strain P2261	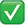	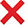	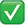	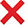	Stool microbiome studies have previously shown that adequate balanced *A. muciniphila* presence is associated with clinical response in patients on ICIs (26, 71–74). Preliminary phase 1 data for Oncobax-AK in combination with ipilimumab/nivolumab in advanced clear cell RCC shows an ORR of 50% with documented microbiome marker changes. A trial in patients who lack *Akkermansia* spp. on stool testing at baseline and who have an advanced clear cell RCC diagnosis on ipilimumab/nivolumab therapy or NSCLC diagnosis (NCT05865730) is ongoing.
**Prebiotics and live biotherapeutic products**					
**Prebiotics and foods containing substances with defined or potential prebiotic effects**					
*Faecalibacterium prausnitzii*, strain EXL01	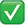	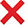	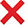	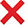	Stool microbiome studies have previously shown that high levels of *F. prausnitzii* is associated with clinical response in patients on ICIs (75, 76). Several studies are ongoing, including a multicenter phase II study in patients with PD-L1 CPS ≥ 5 with advanced gastric cancer receiving nivolumab and FOLFOX in combination with EXL01 (NCT06253611); pilot phase I/II study of the combination of EXL01 with nivolumab treatment for NSCLC patients refractory to immunotherapy (NCT06448572, EXLIBRIS); and addition of EXL01 to atezolizumab-bevacizumab for advanced HCC patients refractory to first-line treatment (NCT06551272, MOTHER).
*Lactococcus lactis*, strain GEN-001	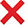	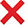	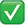	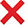	*L. lactis* is transient member of the human gastrointestinal microbiome, typically ingested through fermented foods. Interim results from phase II (NCT05419362) for a combination of GEN-001 with avelumab for advanced gastric/gastroesophageal junction (GEJ) cancer, showed positive antitumor activity with a promising objective response rate (ORR) of 37.5% in patients previously treated with immunotherapy. Publication of completed NCT05419362 results is awaited, as well the ongoing current phase II NCT05998447 for GEN-001 in combination with pembrolizumab ± mFOLFOX for advanced refractory biliary tract cancer.

Ongoing trials of LBP consortia-based combination products, such as VE800, MET-4, VSL#3, Kex02 and BMC128, have been summarized elsewhere (44), with each combination product requiring separate analysis on its own merits due to strain-specific effects. Available postbiotic and synbiotic data is too limited for an adequate summary. Supplemental interventions that currently only have preclinical data, including but not limited to mycotherapy and fucoidan, have not been included. The two ongoing omega-3 trials have not been included due to pleiotropic effects on both the gastrointestinal microbiome and inflammation via the eicosanoid pathway (NCT04965129, NCT04682665), and vitamin D supplementation is not included, having been covered in detail in other literature (2, 6, 65). A green tick denotes that evidence of impact for intervention alongside ICI therapy is available, and a red cross denotes that evidence is not currently available. CPS, combined positive score; DCR, disease control rate; EFS, event-free survival; FMD, fasting mimicking diet; HNSCC, head and neck squamous cell carcinoma; irAE, immunotherapy-related adverse events; NSCLC, non-small cell lung cancer; ORR, objective response rate; OS, overall survival; PFS, progression-free survival; RCC, renal cell carcinoma, TRE, time-restricted eating.

*Other single strain LBPs currently in active trials include *Bifidobacterium animalis* subsp. lactis BL–04, Lactobacillus rhamnosus Probio–M9, *Lactobacillus johnsonii* and R5780.

- *Mediterranean diet*—MINI-MD trial in metastatic melanoma (NCT06236360).
- *Increased fiber intake or supplementation*—DIET-LUNG in NSCLC patients on ICIs increasing fiber intake to at least 25 g daily (NCT05805319); DIET trial at MD Anderson (NCT04645680, 50 g fiber/day from whole foods vs. healthy control diet at 20 g fiber/day) in melanoma patients on ICI (53); FORX01 trial (FOod interventions to Reduce immunotherapy toXicity) supplying food boxes with increased fiber content and 30 different plants per week to patients (NCT05832606).
- *Dietary enrichment with prebiotic-containing foods*—prebiotic food-enriched diet (PreFED) in ICI-refractory melanoma patients on ipilimumab and nivolumab (NCT06250335).
- *Dietary enrichment with fermented foods*—3–6 servings of fermented foods per day in patients with stage II-IV NSCLC and locally advanced rectal cancer (NCT06337552); cross-over trial of fermented foods and fiber supplementation in patients with melanoma and NSCLC (NCT06475807).
- *Fasting regimes*, including intermittent fasting (IF) and time-restricted eating (TRE), and *fasting mimicking diet (FMD)*:
  • Fasting—feasibility study of short-term fasting (STF) for 48 h before immunotherapy and 24 h after immunotherapy in advanced skin cancer (NCT04387084) (54); TRE with 14-h of prolonged nightly fasting in treatment-naïve metastatic head and neck squamous cell cancer (mHNSCC) receiving ICIs (NCT06603155).
  • FMD—FMD-ICI trial examining a 4-day FMD in advanced cancer patients on anti-PD-1/PD-L1 or anti-CTLA4 ICIs, alone or in combination (NCT06438588).
- *Ketogenic diet (KD)*—ketogenic diet in patients with metastatic melanoma and renal cancer (NCT06391099).

While the Mediterranean diet, fiber, prebiotic and fermented foods interventions have a more obvious rationale behind them, fasting and ketogenic diet are perhaps less well understood in their potential synergy with ICIs. The mechanistic rationale for fasting-based interventions seen in preclinical research include but are not limited to tumor microenvironment (TME) immunomodulation and metabolic reprograming, including mTORC1/B7-H3 suppression, with resulting reduced MDSC infiltration, switch to an antitumoral TAM phenotype and enhancement of CD8 T cell cytotoxicity (55–57). Some of the effects of fasting may be mediated through its impact on the gut microbiota, including increasing *Akkermansia muciniphila* levels (58). A preliminary trial report from NCT05083416 studying prolonged nightly fasting (PNF) of 14 h as a type of TRE showed that this intervention was associated with significant improvement in disease control rate (DCR) at 3 and 6 months in treatment naïve metastatic HNSCC patients treated with single agent pembrolizumab (59), and the completion of the NCT06603155 prolonged nightly fasting trial is awaited to better guide clinical practice.

As another intervention in the fasting field, preclinical data on FMD in combination with ICIs showed delayed melanoma growth and reduced cardiotoxicity in murine models (60). Fasting-based strategies may be more feasible to implement than methionine-restricted diets, which have also shown preclinical potential for therapeutic synergy with ICIs (61). However, methionine-restricted diets have several challenges, including long-term adherence issues and care needed to avoid over-restriction, which could lead to anorexia and rapid weight loss (62). Methioninase-based strategies or small molecule-based methionine adenosyltransferase 2α (MAT2A) inhibitors may be more useful to explore in targeting methionine metabolism for ICI synergy in the future.

Moving on to the ketogenic diet, preclinical data shows potential for creating a more immunogenic environment, particularly through effects on CD8 T cells, M1 macrophages and NK cells but no human data is currently available (63). Mechanistic concerns in the community remain around whether the changes in the microbiome induced by the ketogenic diet, particularly the high saturated fat version rather than a prudently formulated MUFA-predominant omega-3 enriched regime, may be unfavorable and limit applicability of preclinical data to human trials (64). One of the ketogenic diet trials (NCT05119010, KETOREIN) in metastatic renal cell carcinoma (mRCC) has been terminated recently due to issues with recruitment because of patient refusal to join this dietary intervention study, competition with another microbiome trial and other logistical challenges, so studies in this area are not without their challenges. Currently there is insufficient data to assess the risk-benefit of a ketogenic diet used alongside ICI therapy, and a prudent clinical approach would rely on existing data supporting a wholefood-based polyphenol-rich dietary pattern, such as the Mediterranean diet, with high fiber intake and reduction in UPF and saturated fat consumption.

With several prospective trials on diverse dietary strategies above scheduled to report within the next 2-3 years, research in this field is likely to yield more detailed guidance for multidisciplinary oncology teams taking care of patients on ICIs going forward. It is important to consider proactively how healthcare systems and infrastructure will need to be adjusted to support real-world implementation of nutritional interventions. This may include upskilling medical and nursing teams around baseline nutritional counseling and prompt referrals, increasing capacity for clinical nutrition staff to provide personalized guidance and supervision, and considering the importance of behavior change support, such as health coaching, to deliver sustainable change. Delivering culturally competent nutritional advice with equitable access, including considerations around food security and whole food provision schemes, should form an important part of this agenda to deliver practical impact for patients.

### 2.2 Prebiotics, live biotherapeutic products, postbiotics and synbiotics—modulating the microbiome to support ICIs

There has been a recent rapid expansion of trials in live bacterial/biotherapeutic products (LBPs) and to a lesser extent, prebiotics in ICI treatment, with postbiotics and synbiotics receiving comparatively less attention (see Table 1 for available evidence summary). A word of caution could be offered in the case of LBP interventions in the need for assessment of baseline microbiota, which has already been incorporated in several trials. As an example, the beneficial level for certain species is likely to lie within a specific range, which was illustrated for *Akkermansia muciniphila* with a 10% increase in objective response rate (ORR) to ICIs, provided there was < 4.8% threshold of relative abundance, with both high and low levels associated with poorer outcomes (44, 71). Therefore, simply adding an LBP that may not match the individual’s microbial ecology is unlikely to yield optimal outcomes. Influence of co-existing species should also be considered, both helpful commensals, such as *Eubacterium hallii* and *Bifidobacterium adolescentis* as companions for *A. muciniphila* (71), and more dysbiotic species, such as increased Proteobacteria and *Veillonella* that have been associated with colitis as an irAE. Strategies to tackle dysbiotic species without antibiotics and with an eye to the preservation of beneficial commensal balance are needed. Microbiome modulation alongside ICI therapeutics may therefore require assessing and addressing the ecosystem more broadly, and clinically any probiotic or prebiotic intervention should be considered for implementation within the prudent dietary pattern discussed above.

#### 2.2.1 Exploring prebiotics for ICI support

According to the latest expanded consensus definition from International Scientific Association for Probiotics and Prebiotics (ISAPP), a prebiotic is a substrate that is selectively utilized by host microorganisms conferring a health benefit (77). Prebiotics must be selectively utilized by an identified body of host microorganisms, must not be degraded by the target host enzymes and have adequate evidence of health benefit for the target host. Examples of typical prebiotics include oligosaccharides, such as inulin, FOS (fructooligosaccharides), GOS (galactooligosaccharides), MOS (mannanoligosaccharide) and XOS (xylooligosaccharide), as well as a broad range of other substances, including human milk oligosaccharides (HMOs) (77).

Trials looking at prebiotic effects alongside ICI treatment include the following categories:

- *Administration of a standard prebiotic*—PRINCESS study of inulin in metastatic HNSCC (NCT05821751), resistant potato starch in patients with solid tumors on dual ICIs (NCT04552418).
- *Administration of candidate prebiotics*, such as omega-3 PUFA (polyunsaturated fatty acid) supplementation (NCT04965129 in NSCLC).
  • It should be clearly noted that due to their well-established anti-inflammatory effects within the eicosanoid pathway long chain omega-3 PUFAs would be expected to have pleiotropic effects independent of microbiome modulation in this setting (78, 79).
- *Administration of foods that contain substances with defined or potential prebiotic effects*—two camu camu trials as a polyphenol-rich supplement in patients with NSCLC, melanoma and mRCC (NCT05303493, NCT06049576).
  • Oral administration of castalagin from camu camu has led to enrichment for bacteria associated with efficient immunotherapeutic responses (Ruminococcaceae and Alistipes) and improved the CD8 + /FOXP3 + CD4 + ratio within the tumor microenvironment (80). Preclinical data show an increase in *Akkermansia muciniphila* with camu camu administration in murine models (81, 82), and two cases of durable deep response in ICI-refractory metastatic melanoma have been documented in the literature, albeit with limited generalizability of these findings (83). Data from prospective trials of camu camu is awaited to guide risk-benefit assessment and any potential impact on clinical practice.

Strangely, there is a notable lack of studies on GOS and partially hydrolysed guar gum (PHGG), both of which have been studied in GI microbiome modulation outside of the ICI setting, including preferentially supporting *Bifidobacteria* spp. growth and healthy SCFA (short chain fatty acid) levels (84–86), which is highly relevant for ICI therapy. Given their low cost, ready availability and ease of administration, future trials incorporating these interventions should be considered. Additionally, further study of targeted interventions with prebiotic effects aimed at correcting baseline microbiota patterns, such as gold kiwi powder to support *Faecalibacterium prausnitzii* levels (87) and resveratrol, pomegranate extract/juice and possibly proanthocyanidin-rich aronia berry or cranberry products to support *Akkermansia muciniphila* (88–92).

#### 2.2.2 Live bacterial/biotherapeutic products in ICI support

LBP trials alongside ICI therapy have been ongoing for some time, and current approaches under study include consortia-based products like MET4, VE800 and BMC128, as well as single strain products, such as CBM588, EDP1503, GEN-001, Probio-M9 and many others (44). Assessment of LBP impact should be clearly strain- and therapy-specific, and any trials should include microbiome assessment at baseline and follow-up to assess for response-associated patterns that may be utilized clinically to enable personalization. Meta-analyses that combine all LBPs without consideration of strain or consortia composition are unhelpful to guide clinical utility and may be likened to amalgamating data for all medications in any medical condition without regard to class, such as all antihypertensives. It is essential that clinicians in this arena understand and treat each probiotic strain or consortium almost as an individual medication with its own indications and risk-benefit within a specific context. For manufactures of LBPs it may be important to note that formulation of such products would ideally avoid excessive excipients, focusing on the active ingredient within a hypoallergenic matrix suitable for a wide variety of patients, such as cellulose- or brown rice powder-based matrix inside an HPMC (hydroxypropyl methylcellulose) capsule where possible.

It is beyond the scope of this review to cover all the LBP trials currently running in combination with ICIs but specific single strain LBP highlights within current research are outlined below (see also Table 1):

- *Clostridium butyricum CBM 588 strain*—butyrate producer, shown to increase the abundance of beneficial bacteria like *Bifidobacteria* spp., enhance the intestinal barrier function, and modulate immune responses by promoting the expansion of specific T cell populations, including IL-17A-producing γδT cells and CD4 + T cells (93, 94)
  ∘    Past research—retrospective analyses show benefit for OS in NSCLC patients receiving chemoimmunotherapy, as well as improved OS for those who have received PPIs or antibiotics (67, 68), making this a potential GI microbiome rehabilitation strategy for patients whose clinical status requires such treatment during ICI therapy. Combination data in metastatic RCC (mRCC) patients treated with cabozantinib and nivolumab showed improved PFS and ORR as a preliminary signal (69, 95), and long-term follow-up presentation at ASCO 2025 for CBM588 with nivolumab/ipilimumab in mRCC showed improved ORR, PFS and OS (70), although full publication is awaited.
  ∘    Ongoing research—CBM588 in mRCC in combination with nivolumab and cabozantinib (NCT05122546), phase I dose escalation trial of CBM588 in combination with nivolumab/ipilimumab in mRCC patients (NCT06399419).
  ∘    Not yet recruiting – assessing risk of recurrence after surgery for RCC patients with CBM588 with pembrolizumab vs. pembrolizumab alone (NCT07037004).
- *Akkermansia muciniphila, strain P2261*
  ∘    Ongoing research—Oncobax-AK in patients who lack *Akkermansia* spp. on stool testing at baseline and who have an advanced clear cell renal cell carcinoma diagnosis on ipilimumab/nivolumab therapy (96) or NSCLC diagnosis (NCT05865730).
- *Faecalibacterium prausnitzii, strain EXL01*
  ∘    Ongoing research—multicenter phase II study in patients with PD-L1 combined positive score (CPS) ≥ 5 with advanced gastric cancer to evaluate the efficacy and safety of nivolumab and FOLFOX in combination with EXL01 as first-line treatment (NCT06253611); pilot phase I/II study of the combination of EXL01 with nivolumab treatment for NSCLC patients refractory to immunotherapy (NCT06448572, EXLIBRIS); addition of EXL01 to atezolizumab-bevacizumab for advanced HCC patients refractory to first-line treatment (NCT06551272, MOTHER)
- *Lactococcus lactis, strain GEN-001*—*L. lactis* is transient member of the human gastrointestinal microbiome, typically ingested through fermented foods.
  ∘    Interim results from phase II (NCT05419362) for a combination of GEN-001 with avelumab for advanced gastric/gastroesophageal junction (GEJ) cancer, showed positive antitumor activity with a promising objective response rate (ORR) of 37.5% in patients previously treated with immunotherapy (97).
  ∘    Ongoing research—phase II study for a combination of GEN-001 with avelumab for advanced gastric/gastroesophageal junction (GEJ) cancer as above (NCT05419362), phase 2 for GEN-001 in combination with pembrolizumab ± mFOLFOX for advanced refractory biliary tract cancer (NCT05998447).

#### 2.2.3 Postbiotics and synbiotics

Postbiotic and synbiotic trials appear to be scarce in ICI combination therapy. This leaves an important gap for further research that could look at rational synbiotic combinations of a variety of interventions, such as camu camu and *Akkermansia muciniphila* strain P2261, EXL01 and golden kiwi powder or Probio-M9 and PHGG. The main synbiotic trial in progress currently is a phase II randomized study (NCT03870607) of the use of pre-and probiotics during the definitive treatment of chemotherapy-radiotherapy (Ch-RT) for patients with localized anal canal squamous cell cancer (ACSCC).

As an example of postbiotic research, one trial of fermented soybean solution MS-20 is ongoing in patients with NSCLC on pembrolizumab (NCT04909034). This fermented soybean solution is made using a unique combination of bacterial strains, including *Lacticaseibacillus paracasei, Lactobacillus delbrueckii subsp. bulgaricus, Bifidobacterium longum*, along with the *Saccharomyces cerevisiae* yeast. The final product does not contain live microorganisms, as they are heat inactivated and removed by filtration, therefore putting this intervention into the postbiotic category (98). Prior work on JK5G as a postbiotic with high concentrations of more than 21 inactivated *Lactobacillus* strains and their metabolites showed that JK5G increased *Faecalibacterium*, *Ruminococcaceae*, and fecal butyrate concentration, and diminished *Escherichia-Shigella*, alongside improving quality of life, adverse events and inflammatory markers in NSCLC patients on anti-PD-1-based chemoimmunotherapy (99). Further research on standardized butyrate-rich postbiotic products is warranted to assess potential contribution to supporting ICI treatment alongside other strategies explored above.

#### 2.2.4 Medications as key influences on microbiota and ICI response

There is a well-established relationship between several classes of medications that affect both the GI microbiota composition and ICI outcomes, particularly PPIs (proton pump inhibitor) and antibiotics, with important antibiotic stewardship guidance produced in a recent ASCO education book for clinicians (44). PPI use leads to oralization of the GI microbiome (100) and is linked to poorer ICI outcomes with a recent meta-analysis showing a 12% higher risk of disease progression and an 18% increased mortality risk with concomitant use (101), as well as increased risk of nephritis and acute kidney injury (102). Therefore, a proactive medical re-assessment and consideration of careful PPI discontinuation may be indicated in preparation for treatment. It is also important that clinicians assess patients for a history of recurrent infections prior to initiation of ICI treatment and consider optimizing factors that promote immune health and minimize antibiotic use in the future to avoid impacting ICI effectiveness, such as:

- optimizing vitamin D levels to at least 75 nmol/l serum 25-hydroxyvitamin D, which has been shown to support immune health and positively impact ICI outcomes (2, 65, 66, 103),
- managing chronic urinary tract infections (UTIs) through proactively addressing vulvovaginal atrophy and considering UTI vaccines, such as sublingual MV140 (104), or
- managing recurrent respiratory tract infections (RTIs) through co-morbidity control and consideration of bacterial lysates, such as OM-85 (105).

Other medications beyond antibiotics, PPIs and steroids may have systemic effects that impact ICI therapy, including laxatives and opioids, with antipsychotics and benzodiazepines under study (44).

### 2.3 Beyond the obvious—looking at nutraceuticals and mycotherapy as potential synergistic approaches

Mycotherapy (use of medicinal mushrooms) should be a key focus for future ICI combination trials as a low-cost intervention with relevant microbiome modulation and immunoregulatory mechanisms (106, 107). Preclinical evidence shows that *Ganoderma lucidum* (reishi mushroom) polysaccharide supplementation alleviated dysbiosis and significantly activated T-cell-mediated antitumor response in combination with anti-PD-1 immunotherapy in colorectal cancer (108). Additionally, preclinical research on AHCC derived from shiitake mushroom (*Lentinula edodes*) also showed a beneficial effect with dual ICI blockade (anti-PD-1 and anti-CTLA-4) in colon cancer with increase in Ruminoccoccaceae and activation of CD8 T cell effector responses with resulting decrease in murine tumor growth (109). Early cell-based data on cordycepin impact on CD8 T cell effector mechanisms and M2 to M1 macrophage reprograming with a potential potentiation of antitumor effect of anti-PD-L1 needs further *in vivo* evaluation (110). With a building body of preclinical evidence, prospective studies of mycotherapy interventions conducted by expert multidisciplinary teams should be actively encouraged.

As an example of another relevant nutraceutical approach, preclinical data for fucoidan shows that combination therapy with anti-PD-1/PD-L1 agents results in metabolic reprograming in breast cancer cells and modulation of TME through SCFA production that improves CD8 T cell effector function and suppresses regulatory T cells to lift immunosuppression (111, 112). Further clinical studies are awaited, and there may be potential for combination strategies, such as co-administration of AHCC and fucoidan with commercial products already available for research.

It is important to note that as with LBPs or prebiotics, in clinical practice (outside of interventional research) any nutraceutical approaches should be provided within the overall prudent fiber- and polyphenol-rich dietary pattern that supports healthy GI ecology, aiming for enhancement of effect and not as a replacement of core lifestyle foundations.

### 2.4 Future directions in nutrition and natural product research for ICI support

**FIGURE 1:**
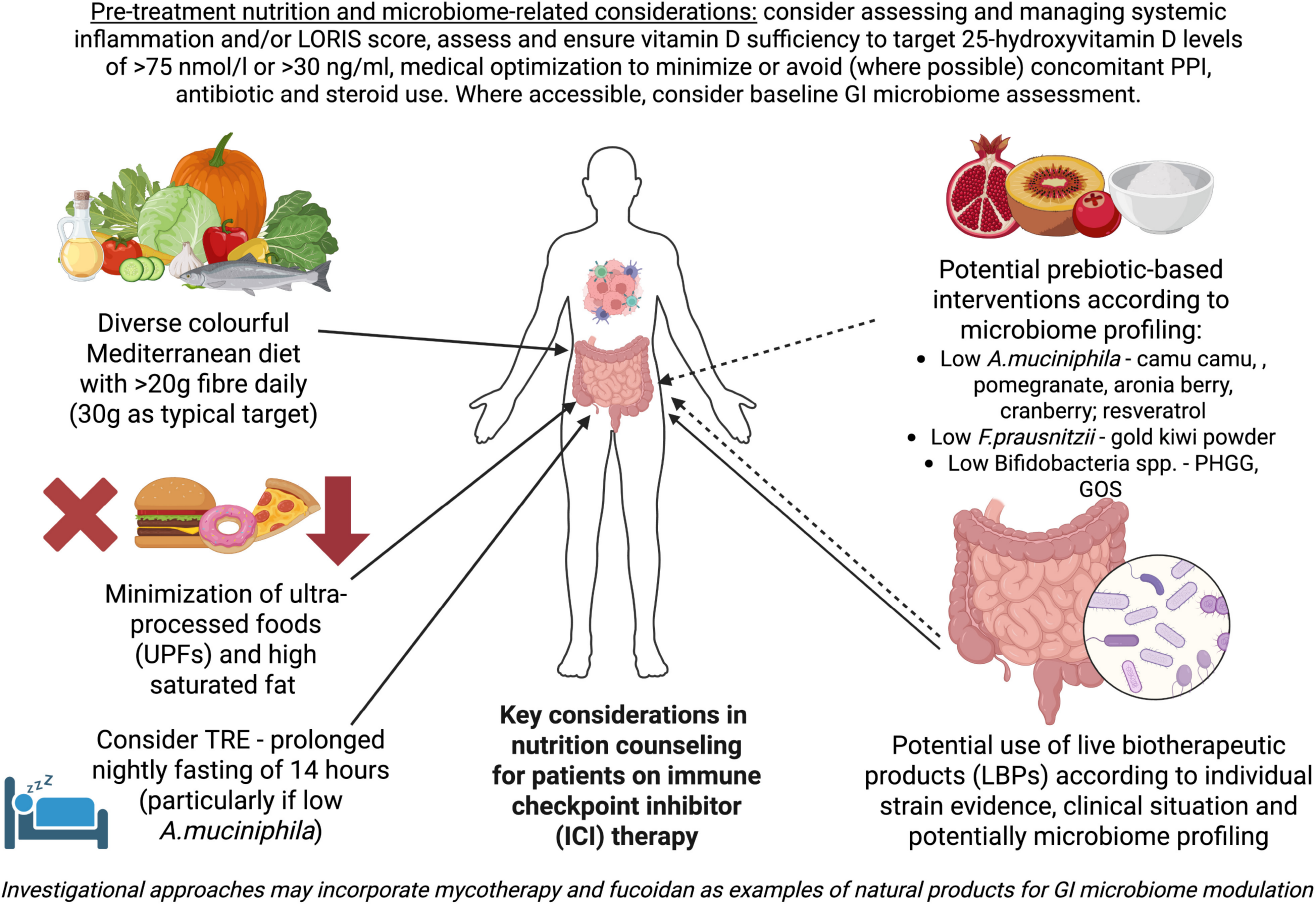
Key considerations in nutrition counseling for patients on immune checkpoint inhibitor (ICI) therapy. Solid arrows highlight data available from current studies in patients on ICIs, mainly alongside anti-PD-1/PD-L1 therapy alone or in combination with anti-CTLA4 agents, which have been outlined in this review and prior work (2). Dashed arrows illustrate potential interventions based on limited human data showing favorable impact on microbial species associated with improved ICI response outside of direct ICI co-treatment studies or preliminary trial reports/case series illustrating potential benefit combined with preclinical data. Created and adapted in BioRender. Fuller-Shavel, N. (2025) https://app.biorender.com/illustrations/687e6d297e19017a3671da71.

As a summary of our current knowledge and potential targeted interventions, Figure 1 provides a starting point for nutrition counseling that will be dynamically changing with emerging trial data. Reports from ongoing studies highlighted above should allow us to elucidate the role of dietary interventions, LBPs and certain prebiotic-based approaches better, although important gaps surprisingly remain in simple prebiotic, postbiotic and synbiotic research. Further studies on mycotherapy and fucoidan, alongside ongoing data from camu camu trials, may provide important information on using foods and nutraceuticals alongside ICIs for synergistic effect, depending on performance shown in appropriately powered prospective trials. In all upcoming research baseline and post-intervention assessment of the GI microbiome will be important to incorporate for assessment of distinct patterns associated with companion intervention response and clinical outcomes for ICI therapy. Using simple biomarker-based scores that predict ICI response, such as established inflammatory indices (2) and LORIS^1^ (113), may become important not just for baseline assessment but in the future as a proactive driver for intervention to potentially modify the patient’s clinical trajectory. A combination of desktop assessments and understanding baseline dysbiosis patterns in individual patients and how to intervene in a targeted manner may offer clinical teams an opportunity for personalized prehabilitation and on-treatment support for patients within a dynamic precision nutrition implementation model.

Going beyond aspects highlighted above, an acknowledgment of important synergistic effect of multimodal interventions that incorporate but are not limited to nutrition and natural products is essential. Exercise is a well-established immunological and GI microbiome modulation intervention (114, 115) that has shown significant clinical impact in the recent adjuvant CHALLENGE trial for patients with resected colon cancer (116). Alongside data from previous small studies on exercise alone (2), here are two important ongoing multimodal trials, including NCT06298734 (DUO Trial in advanced melanoma with high fiber diet and high intensity exercise) and NCT04866810 (plant-based high fiber diet in combination with exercise in melanoma patients). In the meantime, physicians are encouraged to counsel patients to follow standard oncological physical activity guidelines under the supervision of a cancer exercise/rehabilitation professional (117).

## 3 Nutritional approaches aimed at minimizing PI3K inhibitor toxicities

Moving on from immune checkpoint mechanisms, the PI3K/AKT/mTOR pathway plays a central role in regulating glucose homeostasis, lipid biosynthesis, protein synthesis and cell survival, making it a critical axis in both tumor progression and host metabolic balance (118). In breast cancer, targeted inhibition of PI3K/AKT/mTOR pathway using alpelisib, a selective PI3Kα inhibitor, has improved progression-free survival in patients (15). This clinical benefit is accompanied by high rates of hyperglycemia, with up to 64% of patients experiencing elevated fasting glucose levels. The metabolic toxicity is exacerbated in patients with underlying insulin resistance or obesity, highlighting the importance of pre-initiation testing, monitoring and maintaining balanced glucose levels. This is further supported by preclinical studies showing that systemic glucose-insulin feedback can be affected through dietary or pharmacological interventions, significantly improving the efficacy-to-toxicity ratio of PI3K inhibitors (119).

In colorectal cancer (CRC), *PIK3CA* mutations have been shown to reprogram glutamine metabolism by upregulating glutamate pyruvate transaminase 2 (GPT2), thereby increasing tumor cell dependence on glutamine. These findings implicate oncogenic *PIK3CA* mutations as a driver of glutamine dependence in CRC and suggest that targeting glutamine metabolism may represent a viable therapeutic strategy for patients harboring *PIK3CA* mutations (120). Preclinical data suggest that overexpression of the vitamin D receptor (VDR) may interact with the RAS-MAPK and PI3K-AKT pathways in colorectal cancer, supporting the potential for chemoprevention strategies targeting VDR (121). Further research is needed to assess nutritional and nutraceutical strategies that may be able to target these mechanisms, such as EGCG (epigallocatechin gallate) as a potential glutaminase inhibitor (122), and assess potential clinical impact of combination approaches.

From a practical nutritional perspective, patients undergoing treatment with PI3K inhibitors require comprehensive metabolic management to mitigate therapy-related adverse effects. Proactive monitoring of fasting glucose and HbA1c, early introduction of agents, such as metformin and/or SGLT2 inhibitors, and close follow-up during the first weeks of therapy are therefore recommended to mitigate this predictable, mechanism-based adverse effect (123). Key dietary strategies include adherence to a low glycemic index and lower carbohydrate diet, reduction of refined carbohydrate intake, and maintenance of adequate protein consumption to support lean body mass (124), alongside regular exercise. Regular assessment of fasting glucose, HbA1c, and lipid profiles is critical, and coordinated care with endocrinologists and clinical nutrition staff is recommended, particularly for patients who may benefit from insulin-sensitizing agents, alongside nutrition and regular exercise (11, 124–126). Recent preclinical findings also highlight the role of PI3K signaling in regulating arachidonic acid metabolism, revealing a novel metabolic vulnerability that may potentially respond to dietary fat restriction (127), offering a counter perspective to the high fat and low carbohydrate approaches, which warrants further investigation.

While clinical trial results to support specific nutritional interventions in patients receiving PI3K inhibitors are currently lacking, further clarity on nutritional management strategies may be provided in several upcoming study reports, including NCT05090358 looking at the ketogenic diet, low carbohydrate diet and canagliflozin (SGLT2i) impact on alpesilib-induced hyperglycemia in a three-arm design (128). The feasibility of a personalized well-formulated ketogenic diet in stage IV metastatic breast cancer patients during chemotherapy has been recently established in the Keto-CARE trial (129). Additionally, the NCT06463028 Phase 2 multicenter study of the novel combination of sapanisertib and serabelisib (PIKTOR) with paclitaxel and an insulin-suppressing diet in participants with advanced or recurrent endometrial cancer may also be valuable in furthering our practical toolkit. Future study directions may include multimodal interventions with personalized nutrition and exercise oncology approaches for additional synergies in tackling hyperglycemia with PI3K/AKT/mTOR targeted agents.

## 4 Managing the impact of EGFR-tyrosine kinase inhibitors on nutritional status and gastrointestinal toxicities

EGFR-TKIs are approved as first-line treatments for advanced non-small cell lung cancer (NSCLC) harboring EGFR mutations and have significantly improved both survival and quality of life in affected patients (130).

The nutritional status of patients receiving EGFR-TKI therapy has been evaluated not only as a prognostic indicator prior to treatment but also in terms of how TKIs themselves impact nutritional parameters during therapy. Notably, TKI-associated gastrointestinal toxicity, particularly diarrhea, can further contribute to nutritional deficiencies.

Prior studies examining the association between baseline body mass index (BMI) and survival outcomes in patients treated with EGFR-TKIs have produced conflicting findings. For example, a retrospective cohort study identified low BMI (< 18.5), anemia, and low prognostic nutritional index (PNI) as independent predictors of shorter overall survival in NSCLC patients undergoing EGFR-TKI treatment (131). Similarly, another retrospective analysis demonstrated that underweight patients (BMI < 18.5) had significantly worse progression-free survival (PFS) and overall survival (OS) with EGFR-TKI therapy (132). Additionally, a combination of elevated nutritional risk index (NRI) and systemic inflammation response index (SIRI) has been shown to be significantly associated with poor overall survival (OS) and progression-free survival (PFS) in NSCLC patients, with the combination model offering a valuable novel and potentially modifiable biomarker for supplemental risk stratification in NSCLC patients (133). However, an additional study found that patients with a BMI ≤ 20.8 experienced the longest PFS, although no cohort with BMI < 18.5 was included, and this study assessed only gefitinib (134).

Beyond BMI, weight loss either at diagnosis or during early treatment has emerged as an independent predictor of poorer survival outcomes in EGFR mutant NSCLC patients (135, 136). These observations underscore the importance of thoroughly assessing baseline nutritional status, including weight measurement, and implementing ongoing monitoring and prompt targeted intervention throughout treatment.

Body weight alone may not adequately capture clinically relevant changes in body composition. Sarcopenia, defined as low skeletal muscle mass, has been independently associated with poorer survival and reduced treatment tolerance in lung cancer patients (137). While chemotherapy is known to accelerate muscle loss in cancer patients (138), there is also emerging evidence in the context of EGFR-TKIs. Recent data suggests that, as with chemotherapy, skeletal muscle loss not only occurs during EGFR-TKI therapy, but that early muscle loss may be associated with inferior PFS among patients with lung adenocarcinoma (139). Another study has shown that sarcopenia present before starting treatment predicts worse OS and PFS in patients receiving EGFR-TKIs (140).

These findings are clinically significant because they highlight opportunities for prehabilitation prior to treatment initiation and for supportive rehabilitation during treatment. Personalized nutritional interventions based on anti-inflammatory wholefood-based diets (141, 142) with adequate daily protein intake of at least 1.4 g protein per kg bodyweight (143) alongside structured exercise programs may help mitigate sarcopenia and support normal weight. However, prospective research is needed to confirm whether such strategies improve clinical outcomes alongside TKIs and establish the dose-response relationship for practical guidance.

Gastrointestinal toxicity and diarrhea remain among the most common adverse effects of TKIs, potentially compromising treatment adherence and quality of life. Prompt, proactive management of diarrhea is therefore critical, ideally using a multimodal approach that integrates pharmacologic and nutritional strategies. Oral rehydration therapy (ORT) is recommended for mild diarrhea, aiming to consume at least 30–25 mL/kg/day of beverages containing isosmotic calories at moderate temperatures (144). Dietary modifications should focus on small, frequent meals consisting of easily digestible cooked foods that are low in fat and insoluble fiber (145). Patients should avoid known dietary triggers, including high-fat and fried foods, spicy dishes, insoluble fiber sources, sugar-free beverages, caffeinated drinks, alcohol, and chewing gum, along with herbal or nutritional supplements with laxative effects (146). While probiotics are under investigation for diarrhea management, further randomized trials are required to define optimal strains, dosing, duration, and efficacy in relation to individual microbiota composition and TKI partner therapy.

## 5 Summary and future directions

Nutrition and wider GI microbiome modulation strategies, from prebiotics and live biotherapeutic products and beyond, can play a crucial role in supporting ICI treatment, both in supporting best clinical response for patients and potentially in reducing toxicities. With rising use of ICIs across multiple tumor indications and significant costs for these agents per patient, it is important that multidisciplinary teams are prepared to both utilize available data in nutritional counseling for patients and to keep updated on the emerging data from ongoing clinical trials. Currently the best evidence for dietary support alongside ICIs lies in following a wholefood-based polyphenol-rich dietary pattern, such as the Mediterranean diet, with fiber intake above the minimum of 20 g daily with 30 g as the common government recommended threshold, alongside a reduction in UPF and saturated fat consumption, which is typical of the modern Western diet (2, 25). Ongoing research will elucidate the role of dietary enrichment with fiber, prebiotic-containing and fermented foods, as well as fasting, FMD and ketogenic diet in ICI support.

Alongside nutritional interventions and the importance of optimizing vitamin D levels (2, 66), promising data continues to emerge for camu camu and CBM588 use alongside anti-PD-1 ICIs (67, 70, 80, 83), with many other interventions and combinations being researched. Surprising gaps remain, including poor coverage of synbiotics, postbiotics and common prebiotic interventions that may be targeted at relevant microbial species for ICI response, including PHGG, GOS and gold kiwi powder as some examples. These gaps should be addressed within the field, alongside exploration of mycotherapy and fucoidan co-administration with ICIs as two potential promising additional avenues.

It is likely that future ICI care planning will involve pre-treatment baseline medical review to optimize medications (focusing on avoiding agents associated with poor prognosis, such as antibiotics, PPIs and steroids) and to assess baseline markers, such as vitamin D levels, LORIS and broader measures of systemic inflammation, and where possible, the GI microbiome through TOPOSCORE (original metagenomics-based model or its qPCR-based evolution) or other tools. This may then be followed by a personalized prehabilitation and on-treatment support plan with ongoing dynamic readjustment, particularly where specific trigger events occur, such as administration of antibiotics on-treatment. Other broader support considerations around optimizing ICI therapy, including assessing and addressing chronic stress and exercise oncology recommendations, should also be considered within a multimodal care plan (2).

For PI3K inhibitors the nutritional intervention focus shifts to adherence to a low glycemic index and lower carbohydrate diet, reduction of refined carbohydrate intake, and maintenance of adequate protein consumption to support lean body mass. Regular assessment of glycemic and lipid control is essential with coordinated care with endocrinologists, clinical nutrition and exercise oncology staff to devise personalized lifestyle and pharmacological management plans.

In EGFR-TKI therapy nutritional status and body composition, including BMI and sarcopenia, have been identified as important prognostic factors. Early assessment and continuous monitoring, along with tailored nutritional and exercise interventions, may improve outcomes. Managing TKI-related diarrhea requires proactive multimodal strategies combining diet, hydration, and pharmacological support.

As this review illustrates, there is no “one size fits all” dietary approach to cancer support, and there is a real need to be as specific with nutritional interventions as clinicians are with targeted cancer therapies. Precision nutrition approaches need to involve multidisciplinary team assessment of the patient with analysis of biomarkers and baseline nutritional status, which is then used to design an appropriate dynamic intervention plan, based on their treatment regime, biochemistry and where appropriate, microbiota changes and exposome influences, from medications to chronic stress, that can influence response to treatment (2). Personalized multimodal interventions that include nutrition as a core component may need to start at the prehabilitation stage and be continually modified through the current targeted treatment and beyond into the next phase of survivorship or living well with cancer to achieve better clinical outcomes and quality of life for oncology patients.

## Footnotes

1
https://loris.ccr.cancer.gov/
